# Body Mass Index and Social Media Addiction Among University Students

**DOI:** 10.1192/j.eurpsy.2026.10805

**Published:** 2026-07-31

**Authors:** R. Masmoudi, D. Triki, Y. Mejdoub, F. Cherif, I. Feki, J. Masmoudi, F. Guermazi

**Affiliations:** 1 psychiatry A, Hedi chaker university hospital; 2 preventive medecine, habib bourguiba university hospital, sfax, Tunisia

## Abstract

**Introduction:**

Social media has become common, particularly among young adults. While offering connectivity, its excessive use raises public health concerns, including potential links to physical and mental well-being. This study investigates the relationship between social media addiction and Body Mass Index (BMI) in a sample of Tunisian university students.

**Objectives:**

To examine the relationship between social media (SM) addiction and Body Mass Index (BMI) among Tunisian university students.

**Methods:**

A descriptive and analytical cross-sectional study, conducted among university students in Sfax between January and March 2024. Using an online questionnaire to collect sociodemographic, clinical, and academic data. Social media addiction was assessed using the Bergen Social Media Addiction Scale (BSMAS).

**Results:**

A total of 440 students participated in our study, of which 59.5% were female.

Regarding lifestyle habits, 21.6% were smokers, and 7.7% consumed alcohol. Participants had a leisure activity in 46.1% of cases and practiced sports regularly in 12.7% of cases.

Regarding BMI, we identified that 58.86% of the students had a normal BMI, 14.55% were underweight, 21.82% were overweight, 4.09% were obese, and 0.68% had extreme obesity.

More than half of the students (55%) used social media for more than 4 hours/day. The assessment of social media addiction revealed a median BSMAS score of 17.5, with a high risk of addiction in 20% of the students (n=88).

The analytical study demonstrated that social media addiction was significantly more frequent among those with a BMI ≥ 25 (p<0.001).

**Conclusions:**

The study highlights the need to pay greater attention to the possibly intertwined problems in order to implement effective interventions and preventive measures.

**Disclosure of Interest:**

None Declared

